# Unlocking the Pathological Insights of the Bacterial Infections of Free‐Living Pigeons

**DOI:** 10.1002/vms3.70866

**Published:** 2026-02-27

**Authors:** Ahmed Fotouh, Nady Khairy Elbarbary, Said Elshafae, Sohaila Fathi El‐Hawary, Eman A. Al‐Shahari, Hanan M. Alharbi, Maha A. Aljumaa, Suad Hamdan Almasoudi, Ahmed Ezzat Ahmed, Mohamed Said Diab, Manal Abdullah Mahmoud, Rania Samir Zaki

**Affiliations:** ^1^ Department of Pathology and Clinical Pathology Faculty of Veterinary Medicine New Valley University El Kharga Egypt; ^2^ MBA Marywood University Lackawanna Pennsylvania USA; ^3^ Department of Food Hygiene and Control Faculty of Veterinary Medicine Aswan University Aswan Egypt; ^4^ Department of Pathology Faculty of Veterinary Medicine Benha University Benha Egypt; ^5^ Department of Pediatrics College of Medicine University of Utah Salt Lake City Utah USA; ^6^ Department of Biology Collage of Science Jazan University Jazan Saudi Arabia; ^7^ Health Specialties Basic Sciences and Their Applications Unit Applied College at Muhayil Asir King Khalid University Abha Saudi Arabia; ^8^ Department of Biology College of Science Princess Nourah bint Abdulrahman University Riyadh Saudi Arabia; ^9^ Department of Biology College of Sciences Umm Al‐Qura University Makkah Saudi Arabia; ^10^ Department of Biology College of Science King Khalid University Abha Saudi Arabia; ^11^ Prince Sultan Bin Abdelaziz for Environmental Research and Natural Resources Sustainability Center King Khalid University Abha Saudi Arabia; ^12^ Department of Animal Hygiene and Zoonoses Faculty of Veterinary Medicine New Valley University El Kharga Egypt; ^13^ Department of Animal Hygiene and Environmental Sanitation, Faculty of Veterinary Medicine, Assiut University Assiut Egypt; ^14^ Department of Food Hygiene Safety and Technology Faculty of Veterinary Medicine New Valley University El Kharga Egypt; ^15^ Visiting Scientist Food Science Department Faculty of Agriculutre Purdue University West Lafayette Indiana USA

**Keywords:** bacterial infections, Egypt, pathology, pigeons, poultry farm

## Abstract

Feral birds pose a significant concern to many authors, as they can serve as long‐distance vectors for various microorganisms that may be transmissible to animals and poultry. This study aimed to identify bacterial infections in feral pigeons (*Columba livia* var. *domestica*), their potential role in spreading bacterial pathogens to various Egyptian livestock and the zoonotic significance of this bird species. We conducted the study on 80 healthy feral pigeons, collected from a non‐urban area (Ismailia city) in Egypt during the hunting season from October 2022 to July 2023. We kept the birds in the lab for 72 h, conducting a thorough clinical examination and collecting tissue specimens from various organs of the body. The observed histological lesions were various and numerous, with variable incidences in different body organs. Bacteriological examination revealed the isolation of *Escherichia coli*, *Klebsiella*, *Enterobacter*, *Salmonellae*, *Shigella*, *Proteus*, *Staphylococcus aureus* and *Pseudomonas*. We concluded that feral pigeons could significantly contribute to transmitting some bacterial pathogens to humans, poultry farms and other farm animals.

## Graphical abstract

The potential role of feral pigeons in spreading bacterial pathogens to various livestock and their zoonotic significance. Bacteriological examination revealed the occurrence of *Escherichia coli, Klebsiella, Enterobacter, Salmonellae, Shigella, Proteus, Staphylococcus aureus* and *Pseudomonas*. There were various histological lesions with variable incidences in different body organs.

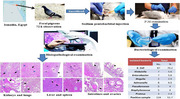

## 1 Introduction

Pigeons are members of the order Columbiformes, which includes pigeons and doves. The common rock dove (*Columba livia*) is the ancestor of all domestic pigeon breeds, with over 800 varieties currently in existence. Pigeons (*C. livia*) inhabit urban, suburban and feral environments (Welle 2021). In many regions, rock doves roost and nest in natural areas but make daily flights of several kilometres to forage in cities and agricultural zones (Welle 2021).

Feral pigeons still constitute a major problem in transmitting a wide spectrum of infectious diseases to domestic animals and humans (Santos et al. 2020). The role of these birds in bacterial transmission is a concern due to their proximity to human habitats and their ability to carry and spread bacteria through their droppings, feathers and others (Dovč et al. 2016). The potential transmission of the disease not only poses a problem in the future and could lead to the emergence of infectious diseases of zoonotic importance, but their exposure to domestic animal diseases could also have severe consequences for their populations (Diab et al. 2019). Different species of feral birds have the potential to spread bacterial microorganisms to domestic birds and animals by nesting and perching close to human activities (Vasconcelos et al. 2018).

The most common bacterial pathogen was *Salmonella*, which can survive in an environment for an extended period. *Escherichia coli* can contaminate feed and water by pigeon droppings (Tanase et al. 2016; Abu El Hammed et al. 2022). Moreover, pigeons were known to carry Campylobacter, causing foodborne illness in humans (Gabriele‐Rivet et al. 2016). As a result, the threat of emerging new infectious diseases is significant (Kobuszewska and Wysok 2024). Nowadays, people are focusing on pigeons due to their significant impact on human health and agricultural production (Soufy et al. 2016). Feral birds are of major concern as they could act as long‐distance vectors for a wide range of microorganisms that are transmissible to humans and animals (El‐Shazly et al. 2016).

Pigeons can also harbour viral (Elmeligy et al. 2024; Shosha et al. 2024) and parasitic agents (Bogach et al. 2021) that are important to domestic birds, as well as to animals and humans. Furthermore, numerous factors contribute to the significance of these birds. Not only can they serve as natural reservoirs for various pathogens, which can then spread to domestic poultry, but they can also transfer infectious agents from poultry products to poultry premises (Fotouh et al. 2020; Zigo et al. 2022). Moreover, feral bird populations can also act as reservoirs of drug‐resistant bacterial pathogens or resistant genetic elements (Fotouh et al. 2024).

Feral pigeons can be challenging due to their adaptability to urban environments and large populations in some areas (Abdel‐Maguid et al. 2019; Santos et al. 2020). Combining multiple control methods tailored to the specific circumstances of each location is often the most effective approach to managing feral pigeon populations and mitigating associated risks such as disease transmission and property damage (Mahmoud et al. 2020; Santos et al. 2020). Addressing the dangers associated with feral pigeons requires a combination of prevention, control measures and public education. Implementing strategies to discourage the presence of pigeons, manage food sources, maintain clean environments and seek professional assistance for population control can help mitigate the risks and dangers posed by feral pigeon populations in urban settings (Chrobak‐Chmiel et al. 2021; Salah et al. 2025).

As there is a lack of reports about bacterial infections in feral pigeons, the present study aimed to identify various bacteria that could be found in pigeons with a trial to illustrate their potential role in transmitting to various livestock as well as their potential role in spreading some zoonotic pathogens in Egypt.

## 2 Materials and Methods

### 2.1 Pigeons

Eighty feral pigeons (*C. livia domestica*) were collected randomly from non‐urban areas in Ismailia, Egypt. The study starts from October 2022 to July 2023. The birds were live‐trapped through nets.

### 2.2 Study Design

Birds were transferred to the laboratory of the Faculty of Veterinary Medicine, New Valley University. Birds were observed for 72 h for the detection of any abnormal clinical signs. All birds were sacrificed using an overdose of sodium pentobarbital (SIGMA, catalogue no. 57‐133‐0) (50 mg/kg body weight). A necropsy examination was carried out, and organs, including the lungs, heart, liver, kidneys, spleen, small and large intestine, pancreas, bursa, brain and pectoral muscle, were dissected out of the body. Specimens from the lungs, liver and intestine were collected under aseptic conditions and kept frozen for further bacteriologic investigations (Al‐Noayme and Al‐Alhially 2021).

### 2.3 Bacteriological Examination

Organs used for bacteriological examination included the liver, lungs and intestines of all birds. All samples were inoculated into Trypticase soya broth and incubated at 37°C for 24 h (Elbarbary et al. 2023).

### 2.4 Isolation of Enterobacteriaceae

A loopful from the previously inoculated broth was cultured onto MacConkey agar and eosin methylene blue agar (EMB) (Oxoid). The inoculated plates were incubated aerobically at 37°C for 24 h, and suspected colonies were picked up and examined for their morphological, cultural and biochemical characteristics (Dutta et al. 2013).

### 2.5 Serological Identification of *E. coli*

The slide agglutination method was used to perform the serological identification of *E. coli* isolates, as described by Abu El Hammed et al. (2022). On a glass slide, the bacterial cultures were mixed with specific antisera and gently agitated to facilitate agglutination. The identity of the isolates was confirmed by the presence of agglutination, which indicated a positive reaction (Dandrawy et al. 2025).

### 2.6 RapIDTM ONE (Remel) Test

It was carried out for the identification of the biochemical profile of the isolated organisms belonging to members of the family Enterobacteriaceae and oxidase‐negative, Gram‐negative non‐fermenters bacteria, according to the manufacturer's instructions.

### 2.7 Isolation and Identification of *Salmonella* spp

Pre‐enrichment in non‐selective liquid medium: Buffered peptone water (10 mL) was inoculated with the test portion (1 g) sample and then incubated at 37°C for 18 ± 2 h. Enrichment in selective liquid media: Rappaport‐Vassiliadis medium with soya (RVS 146 broth) was inoculated with 0.1 mL of pre‐enrichment broth and incubated at 41.5°C ± 1°C for 24 ± 3 h (Khairy et al. 2024). Suspected *Salmonella* isolates were identified serologically using slide agglutination tests in laboratories of the Ministry of Public Health in Cairo, according to Al‐Aalim (2017).

#### 2.7.1 Plating Out and Identification

The xylose lysine deoxycholate agar (XLD agar) was incubated at 37°C ± 1°C and examined after 24 ± 3 h.

### 2.8 Isolation of *Pseudomonas aeruginosa*

A loop‐full from the previously incubated broth was inoculated on Pseudomonas CN selective supplement agar base (Oxoid) and incubated at 37°C for 24–48 h. Suspected colonies were picked up and transferred to a tryptic soy agar slant for further microscopic and biochemical identification (El‐Hawary et al. 2025). Biochemical identification of *Pseudomonas aeruginosa* using API 20NE (analytical profile index) (Bio‐Mérieux, France) was carried out according to the manufacturer's pamphlet (Sambrook et al. 1989).

### 2.9 Isolation of *Staphylococcus aureus*

The samples were incubated at 37°C in thioglycolate broth for 24 h. Then loopfuls from the thioglycolate broth were streaked on the Baird‐Parker agar medium (Oxoid). The inoculated media were incubated at 37°C for 24 h. *Staphylococcus aureus* appeared 2–3 mm, black, shiny and convex and surrounded by clear zones (Fotouh et al. 2014).

### 2.10 Isolation of *Proteus* spp

The samples were incubated overnight at 37°C in the nutrient broth. Then loopfuls from inoculated broth were streaked on MacConkey's agar medium. The inoculated media were incubated at 37°C for 24 h. A few colourless colonies were inoculated into TSI agar, and red slant/yellow bottom, gas and H_2_S production were observed. Moreover, the urease test was positive (Fotouh et al. 2014).

### 2.11 Histopathological Findings

Formalin‐fixed specimens (liver, lungs, intestine and kidneys) were washed in tap water, dehydrated in a graded series of alcohol, cleared in xylene and finally embedded in paraffin (Abo‐Aziza et al. 2022). Paraffin blocks were serially sectioned at 4–5 µm using a microtome (MicroTech_CUT 4050, Germany) and were stained with haematoxylin and eosin (H&E) as routine stains. Sections were examined using a light microscope (Leica DM500, Germany) (Elbarbary et al. 2024).

## 3 Results

### 3.1 Clinical and Post‐Mortem Examination

All birds were healthy; no abnormal lesions could be detected clinically. The observed lesions on post‐mortem examination were not specific. Generally, congestion and abnormal focal lesions were observed in the lungs and liver of some birds.

### 3.2 Bacteriological Examination

Bacteriological examination of the 240 samples from the liver, lung and intestine revealed that 124 were positive for bacterial isolation, with an incidence of 51.7%. Accordingly, the study analysis found that 70.2% (87 samples) tested positive for *E. coli*. The highest prevalence was observed in the intestine (48.7%), followed by the liver and lungs (43.7% and 16.2%, respectively). The lowest prevalence was found in the *Shigella* (2.5%) and *Proteus* (2.4%) samples, as shown in Table 1.

**TABLE 1:** Incidence of isolated bacteria from different organs of the examined pigeon (*n* = 80).

	Examined organ							
Isolated bacteria	Liver		Lung		Intestine		Total	
	No.	%	No.	%	No.	%	No.	%
*Escherichia coli*	35	43.7	13	16.2	39	48.7	87	70.2
*Klebsiella*	3	3.7	1	1.2	3	3.7	7	5.6
*Enterobacter*	2	2.5	0	0	5	6.2	7	5.6
*Shigella*	1	1.2	0	0	1	1.2	2	2.5
*Salmonella*	3	3.7	2	2.5	3	3.7	8	6.5
*Pseudomonas*	2	2.5	3	3.7	0	0	5	4
*Staphylococcus*	3	3.7	2	2.5	0	0	5	4
*Proteus*	0	0	1	1.2	2	2.5	3	2.4
Positive sample	49	20.4	22	9.2	53	22.1	124	51.7

Further analysis of the *E. coli* isolates revealed a diverse range of serotypes. A total of 89 *E. coli* isolates were recovered from various tissue specimens of pigeons, and their serotypes were determined. The results are presented in Table 2. The most common serotype identified was O78, which was isolated from the liver and accounted for 42.5% of the total isolates. The O47 serotype was the second most prevalent, isolated from the intestine and representing 26.5% of the total isolates. The O27 serotype was isolated from lungs and accounted for 17.3% of the total isolates. Other serotypes identified included O149 and O166, each accounting for 4.5% and 3.5% of the total isolates. Five isolates from the intestine (5.7%) remained untyped.

**TABLE 2:** Serotyping of *Escherichia coli* isolated from examined tissue pigeons’ specimens and their detected numbers.

O serotype	Types of tissues	No. of isolates	Percentage of isolation
O78	Liver	37	42.5
O47	Intestine	23	26.5
O27	Lung	15	17.3
O149	Liver	4	4.5
O166	Lung	3	3.5
O untyped	Intestine	5	5.7
Total	—	87	100

**FIGURE 1:**
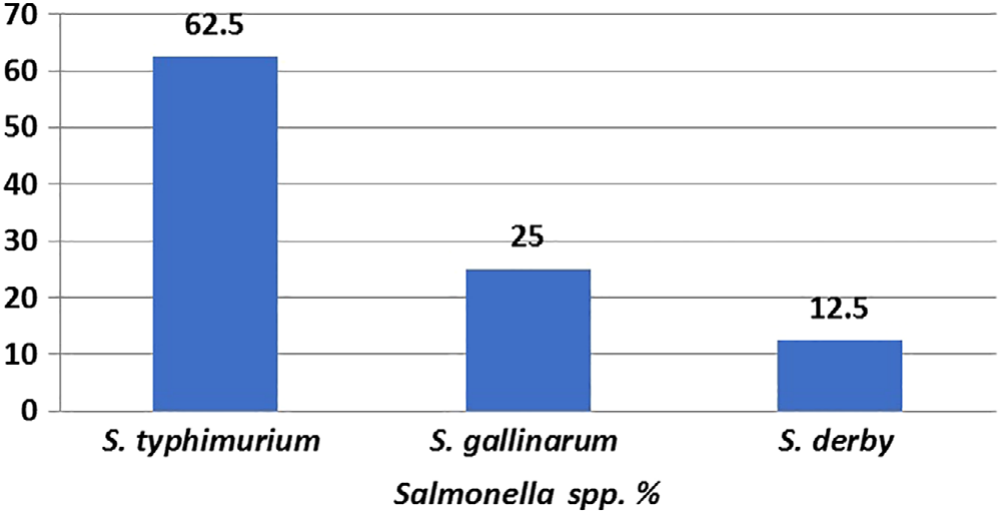
Serotyping of the isolated *Salmonella* spp.

Moreover, the presented study found that 6.5% (eight samples) tested positive for *Salmonella*. Serotyping of the isolated *Salmonella* revealed that 62.5% was *S. typhimurium* (five samples), 25% was *S. gallinarum* (two samples), and 12.5% was *S. derby* (one sample). The results of the various isolated bacteria from different organs, with their incidence, are summarized in Figure 1.

### 3.3 Histopathological Examination

**FIGURE 2:**
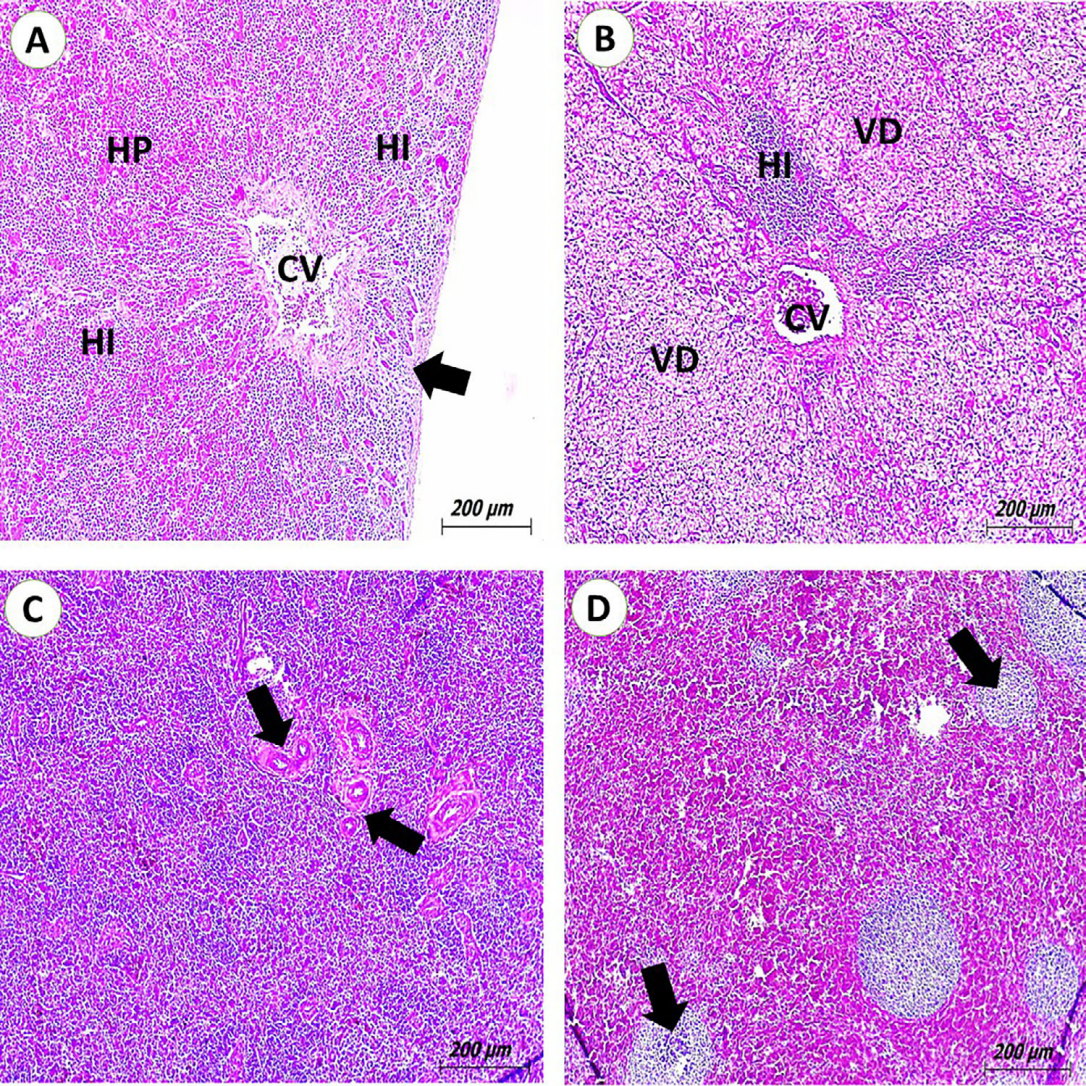
Photomicrograph of pigeon's liver and spleen (H&E scale bar, 200). (A) Liver of pigeons infected with *Escherichia coli* showing perihepatitis (arrow) and massive heterophilic infiltration (HI) of hepatic parenchyma (HP), especially around congested central veins (CV). (B) Pigeon livers infected with *Salmonellae* showing severe vacuolar degeneration (VD) of HP and paracentral HI. (C) Spleen of pigeons infected with *E. coli* showing newly formed splenic arterioles (arrows). (D) Spleen of pigeons infected with *E. coli* showing lymphoid depletion (arrows).

The microscopic lesions observed in the livers of pigeons infected with *E. coli* can vary depending on the severity of the infection and the bird's immune response. Microscopically, we can observe hepatitis, characterized by the infiltration of heterophilic cells and disruption of normal liver architecture. The inflammatory response to the infection manifests as swelling and congestion of the liver's blood vessels. In *Salmonella*‐infected cases, there was vacuolar degeneration (VD), and areas of necrosis within the liver tissue may be present, indicating damage (Figure 2A,B). Lymphocytic depletion was the most commonly observed lesion in the examined spleens infected with *E. coli*. The inflammatory process led to the detection of newly formed blood vessels (Figure 2C,D).

**FIGURE 3:**
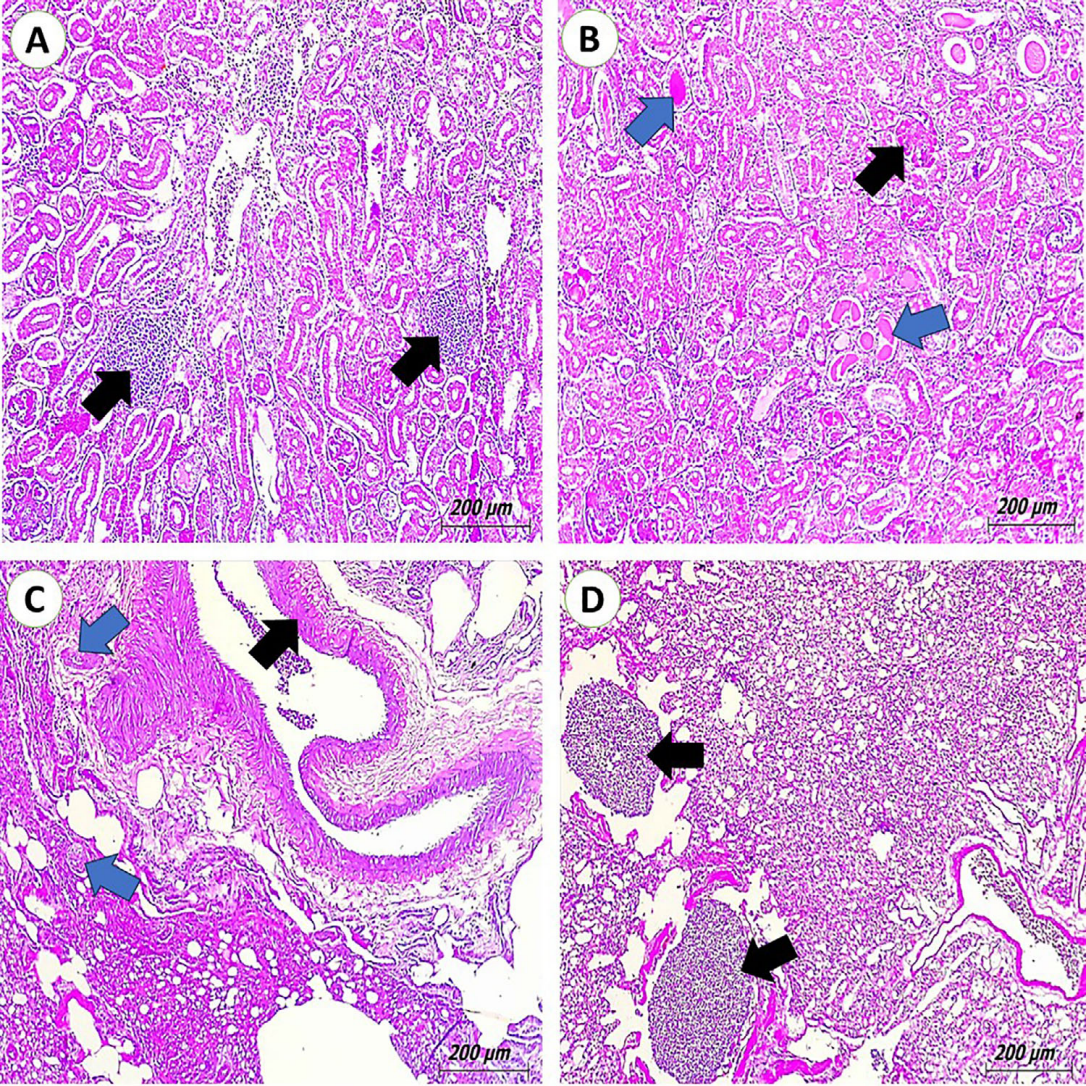
Photomicrograph of pigeon's kidneys and lungs (H&E scale bar, 200). (A) Kidneys of pigeons infected with *Salmonellae* showing interstitial nephritis (arrows). The kidneys of pigeons that had an *Escherichia coli* infection show hyalinization of the glomerulus (black arrow) and renal casts in the convoluted tubules (blue arrows). (C) The lungs of pigeons infected with *Pseudomonas* show congested vasculature (blue arrows) and hyperplasia of the bronchiolar lining epithelium (black arrow). (D) Lungs of pigeons infected with Staphylococcus showing massive infiltration of parabronchus by inflammatory cells (black arrows).

The kidneys showed signs of interstitial nephritis, a condition where inflammatory cell infiltrate the interstitial tissue, causing inflammation. The inflammatory response to the infection resulted in congestion, blood vessel dilation and haemorrhage. The glomerulus and renal casts in convoluted tubules mostly exhibit hyalinization (Figure 3A,B). *Pseudomonas*‐infected pigeons’ lungs show congested vasculature and hyperplasia of the bronchiolar lining epithelium, whereas *S. aureus*‐infected pigeons’ lungs show massive infiltration of the bronchi, lung parenchyma and parabronchus by inflammatory cells (Figure 3C,D). As a result of the infection, there was an accumulation of fluid in the lung tissue due to increased vascular permeability.

**FIGURE 4:**
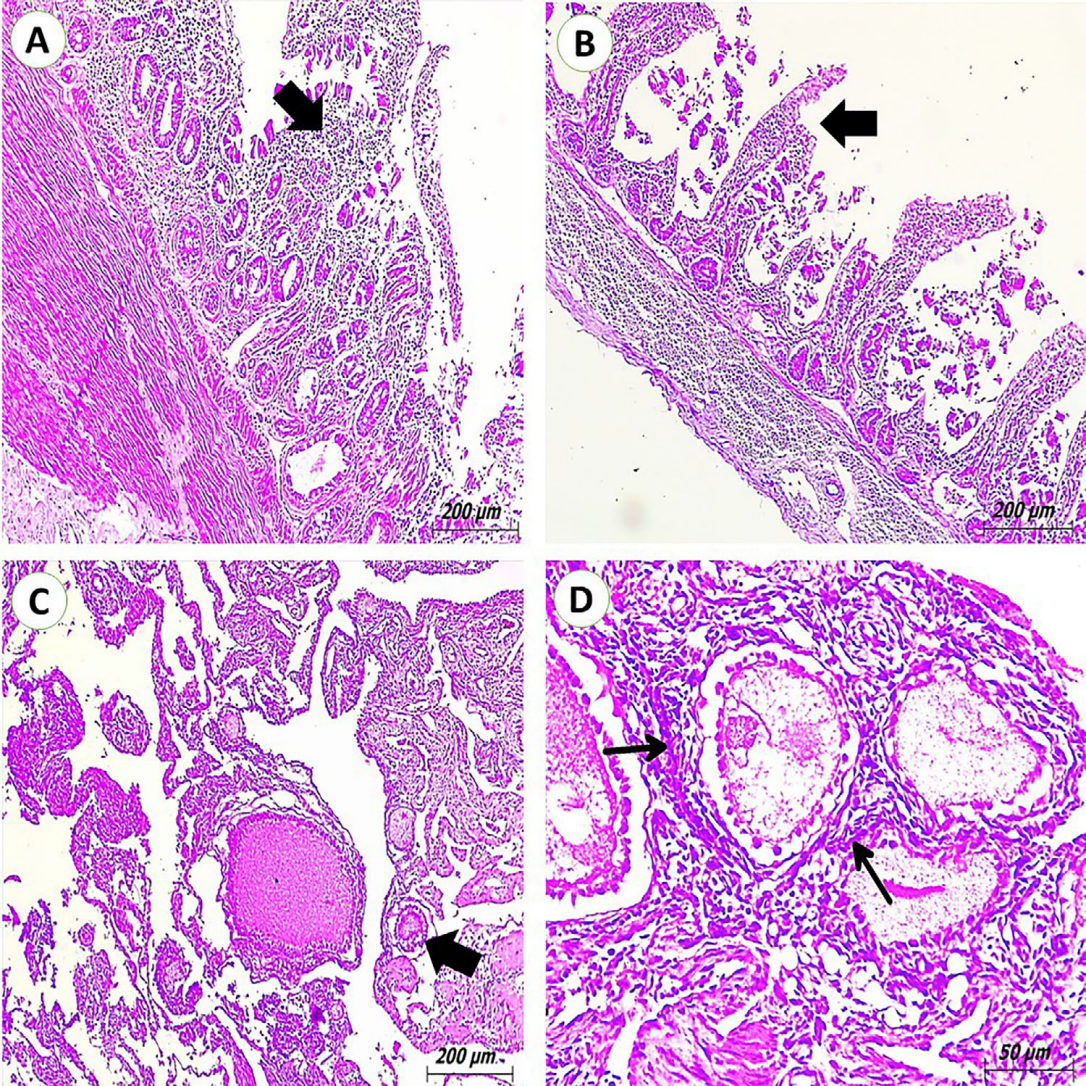
Photomicrograph of pigeon's intestines and ovaries. Infection of pigeons’ intestines with *Salmonellae* causes enteritis, which is shown by inflammatory cell infiltration (arrow) and the breakdown of intestinal glands (H&E scale bar, 200). (B) Intestine of pigeons infected with *Enterobacter* showing desquamation of intestinal villi (arrow) (H&E scale bar, 200). (C) Ovary of pigeons infected with *Salmonellae* showing regressed ovarian follicles (arrow) (H&E scale bar, 200). (D) Ovary of pigeons infected with *Escherichia coli* showing infiltration of ovarian interstitium by inflammatory cells (arrows) (H&E scale bar, 50).

Congestion results from increased blood flow to the intestines. *Salmonella* infections have been known to cause enteritis, characterized by the infiltration of inflammatory cells and damage to the mucosal layer. *Enterobacter* infection causes damage to the intestinal mucosa, resulting in erosions and ulcers in the intestinal lining (Figure 4A,B). In response to the infection, inflammatory cells accumulate in the ovarian tissue, contributing to inflammation and tissue damage in the ovaries. *Salmonella*‐infected ovarian tissues show regressed follicles (Figure 4C,D).

## 4 Discussion

Few studies have surveyed bacterial lesions in feral pigeons in Egypt. The present study was conducted on 80 wild pigeons from Ismailia in the east of Egypt, evaluating various pathological lesions in these birds through a trial to explore their potential role in the epidemiology of certain bacterial pathogens in Egypt. Various body organs, including the lungs, kidneys, intestines, spleen, liver, heart and ovaries, were collected from each bird for histopathological and bacteriological investigation. Furthermore, during 72 h of clinical observation in the laboratory, no abnormal signs were detected in any of the collected birds, but variable histopathological lesions were observed in all of them. Regarding the bacteriological isolation, the main isolates were *E. coli*, *Salmonella*, *Klebsiella*, *Enterobacter*, *Pseudomonas*, *Staphylococcus*, *Proteus* and *Shigella*.

The bacteriological examination of feral pigeons in this study revealed a significant prevalence of 51.7% (124 out of 240) of the collected samples testing positive for at least one bacterial species. This finding underscores the role of free‐living pigeons as important reservoirs and potential vectors for pathogenic bacteria that may pose threats to public health and livestock industries (Diab et al. 2019).

Furthermore, Schmidt et al. (2000) and Radimersky et al. (2010) isolated *E. coli* from feral pigeons, whereas Raue et al. (2005) proved the isolation of *Klebsiella* from pigeons. Additionally, Askar et al. (2011) and Santos et al. (2020) mentioned the isolation of *Shigella* from feral pigeons. As well as El‐Enbaawy et al. (2014) stated that *Pseudomonas* was the most common bacterium in feral pigeons. Awad‐Alla et al. (2010) isolated *Enterobacter* from white ibis in Egypt. The co‐existence of *E. coli*, *Salmonella* and Enterobacteriaceae in pigeons poses a significant threat to human health, as these pathogens can contaminate meat and eggs, leading to foodborne illnesses (Zaki and Hadad 2019).

*E. coli*, a common infectious bacterial disease, was isolated from cases of fibrinous bronchopneumonia, bronchitis and interstitial pneumonia. This result aligns with the findings of Radimersky et al. (2010) and Zaki et al. (2025), who noted that *E. coli* is commonly found in the gastrointestinal tract of birds and is widely disseminated in their faeces. As a result, birds are constantly exposed to contaminated faeces, water, dust and the environment (Charlton 2006; Santos et al. 2020).

In addition, *E. coli* has a role in decreasing immunity, which increases the virulence of the other associated pathogens as well as the ability of the body to produce fibrin in an inflammatory response; it also can cause respiratory manifestations in birds (Bradbury and Janet 2008). The isolated *E. coli* could contribute to hepatitis occurrence (Ewers et al. 2004). Researchers have identified over 1000 *E. coli* serotypes but have only linked a limited subset to avian diseases. The primary reservoir of *E. coli* is the poultry's intestinal tract, where it is present in high concentrations (Diab et al. 2021). Finding the O78 serogroups in the current study samples is worrying because it can be spread from animals to people and is linked to many diseases in humans, such as invasive infections, neonatal meningitis and sepsis (Rahman et al. 2020). Our study revealed that approximately 5.7% of the isolates remained untyped, highlighting the complexity of *E. coli* serotyping and the need for further characterization methods.

El‐Enbaawy et al. (2014) reported that *Pseudomonas* spp. was the most common bacterium in feral pigeons, causing respiratory infections when introduced into the tissues of susceptible birds (Durrani et al. 2020). However, Yousseff and Ahmed (2013) reported that *Pseudomonas* causes interstitial nephritis, deposits urea in the ureters and causes the ureter cells to desquamate. Mohamed and Shehata (2009) observed similar lesions, including interstitial nephritis, degeneration and necrosis of renal tubular epithelial lining cells, in table‐egg layers infected with *Pseudomonas* species. Moreover, *Klebsiella* species are one of the most important zoonotic microorganisms, and the infections they cause are often resistant to multiple drugs. More and more strains are making extended‐spectrum beta‐lactamases (ESBLs), which make the body resistant to many antimicrobial drugs (Beceiro et al. 2013).

*Shigella*, one of the most important foodborne and waterborne diseases, can spread to humans through various routes such as contaminated water from wild bird faeces (Abbaszadegan et al. 1997) or improperly cooked meat (Todd et al. 2007). Furthermore, studies have reported that *Shigella* can cause focal hepatitis and focal hepatic necrosis in both chicken embryos (Al‐Sadi et al. 2000) and humans, particularly laboratory workers (Stern and Gitnick 1976). The predominant lesion in the immune organs (spleen, bursa of Fabricius and caecal tonsils) was lymphocytic depletion, which could be attributed to the isolated *Salmonella* that causes enlargement of the lymphoid organs at the beginning of the infection, followed by lymphocytic depletion and decreased bird immunity, which is in agreement with Ranjbar et al. (2020). The observed post‐mortem lesions were variable and not specific, with no conspicuous lesion associated with a particular locality.

Collectively, these bacteriological findings demonstrate that feral pigeons can harbour a wide array of pathogenic and opportunistic bacteria, many of which have zoonotic or veterinary significance. Their free‐ranging behaviour, ability to thrive in diverse habitats and proximity to urban and agricultural environments make them potential bridge hosts for pathogen transmission between wildlife, domestic animals and humans.

The histopathological findings observed in the tissues of feral pigeons in this study provide valuable insights into the pathological impact of various bacterial pathogens and highlight the potential of these birds as reservoirs for zoonotic and economically important infections. Liver lesions in *E. coli*‐infected pigeons were characterized by hepatitis with infiltration of heterophilic cells and marked disruption of hepatic architecture. These findings are consistent with bacterial septicaemia, where the liver acts as a filtration organ, accumulating bacteria and initiating a robust inflammatory response. Hepatic congestion and swollen blood vessels are hallmarks of acute inflammation, often reported in systemic *E. coli* infections in birds and mammals. Similar histopathological changes have been described by Mehmood et al. (2021), where hepatic inflammation was attributed to circulating endotoxins and bacterial antigens in avian species.

In pigeons infected with *Salmonella*, VD and necrosis within hepatic tissue were evident, suggesting more severe cellular injury. These lesions reflect hepatocellular damage due to the cytotoxins produced by *Salmonella* spp., especially *S. typhimurium*. This pathogen is known for its invasive nature and its ability to induce both acute and chronic granulomatous hepatitis in birds, as previously reported in broilers by Soufy et al. (2016).

Splenic lymphoid depletion, as seen in *E. coli*‐infected pigeons, indicates a depressed immune status and reflects systemic involvement. Lymphoid atrophy is a common feature in chronic infections and stress‐related immunosuppression. The neovascularization observed suggests chronic inflammation and tissue remodelling. These findings align with previous studies that described depletion of splenic white pulp in bacterial infections as a response to persistent antigenic stimulation (Abu El Hammed et al. 2022).

In the kidneys, *E. coli* and *Pseudomonas* infections were associated with interstitial nephritis, congestion and hyalinization of the glomeruli. These changes reflect the systemic dissemination of bacteria and the kidneys’ role in filtering blood‐borne pathogens. Interstitial nephritis with haemorrhage is a known outcome of bacterial endotoxemia and can severely impair renal function. The presence of hyaline casts suggests tubular damage and protein leakage, consistent with findings in septicaemic birds (Fotouh et al. 2014).

The lungs exhibited pathogen‐specific lesions. *P. aeruginosa* infection resulted in bronchiolar epithelial hyperplasia and vascular congestion, indicative of chronic irritation and prolonged inflammation. This bacterium is known for producing biofilms and toxins that damage respiratory epithelium, contributing to tissue remodelling. Meanwhile, *S. aureus* caused massive infiltration of the pulmonary parenchyma and parabronchus by inflammatory cells, a hallmark of bronchopneumonia (Chrobak‐Chmiel et al. 2024). The presence of pulmonary oedema further supports a severe inflammatory reaction leading to increased vascular permeability. These findings are supported by research in avian respiratory diseases where *S. aureus* was found to induce severe fibrinopurulent pneumonia (Mohamed and Abdelaziz 2023).

Intestinal lesions, particularly in *Salmonella*‐infected pigeons, included enteritis with mucosal erosion, inflammation and vascular congestion. These pathological features are indicative of invasive enteric infection, where disruption of epithelial integrity allows for bacterial translocation and systemic spread. *Enterobacter*‐associated ulceration and mucosal damage underscore its role as an opportunistic pathogen in compromised hosts. These findings are consistent with studies showing that both *Salmonella* and *Enterobacter* can cause necrotizing enteritis and colitis in birds, often exacerbated by co‐infections and stress (Mahmoud et al. 2025).

Finally, the ovarian tissue in *Salmonella*‐infected pigeons displayed regressed follicles and inflammatory infiltration, suggesting reproductive dysfunction. *S. gallinarum* in particular is well‐known for its tropism for reproductive tissues in birds (Soufy et al. 2016), often leading to follicular degeneration and decreased egg production in laying hens. The inflammation observed here could result in long‐term impairment of reproductive performance if such infections persist or become widespread in feral populations that interact with poultry (Căpriță et al. 2024).

## 5 Conclusion

In conclusion, the present study revealed a high incidence of bacterial diseases in feral birds without obvious clinical manifestation in the affected birds, suggesting a health risk to humans and animals through direct or indirect contact.

## Author Contributions

**Ahmed Fotouh, Nady Khairy Elbarbary**, **Said Elshafae** and **Rania Samir Zaki**: design of methodology and coordination of the overall study. **Sohaila Fathi El‐Hawary**: data collection and conducted the study. **Eman A. Al‐Shahari**, **Hanan M. Alharbi**, **Maha A. Aljumaa**, **Suad Hamdan Almasoudi** and **Ahmed Ezzat Ahmed**: data collection, data analysis and interpretation of the results. **Ahmed Fotouh**, **Mohamed Said Diab** and **Manal Abdullah Mahmoud**: writing up of the manuscript. **Ahmed Fotouh** and **Nady Khairy Elbarbary**: supervision. All the authors approved the final manuscript for submission.

## Funding

The authors extend their appreciation to the Deanship of Research and Graduate Studies at King Khalid University for funding this work through Large Research Project under grant number RGP2/223/46. The authors extend their appreciation to Princess Nourah bint Abdulrahman University Researchers Supporting Project number (PNURSP2026R454), Princess Nourah bint Abdulrahman University, Riyadh, Saudi Arabia.

## Ethics Statement

The ethical committee for animal use at Faculty of Veterinary Medicine, Aswan University, Egypt, approved the protocol under no. 05‐08‐2022.

## Conflicts of Interest

The authors declare no conflicts of interest.

## Data Availability

All data supporting the findings of this study are available within the manuscript.
